# Effect of COVID-19 Pandemic on Invasive Pneumococcal Disease in Children, Catalonia, Spain

**DOI:** 10.3201/eid2811.211741

**Published:** 2022-11

**Authors:** Pilar Ciruela, Núria Soldevila, Juan José García-Garcia, Sebastià González-Peris, Alvaro Díaz-Conradi, Alba Redin, Belén Viñado, Conchita Izquierdo, Carmen Muñoz-Almagro, Angela Domínguez, Barcino Working Group

**Affiliations:** CIBER de Epidemiologia y Salud Pública, Madrid, Spain (P. Ciruela, N. Soldevila, J.J. García-García, C. Muñoz-Almagro, A. Dominguez);; Agència de Salut Publica de Catalunya, Barcelona, Spain (P. Ciruela, C. Izquierdo);; Universitat de Barcelona, Barcelona (N. Soldevila, J.J. García-García, A. Dominguez);; Hospital Sant Joan de Déu, Esplugues de Llobregat, Spain (J.J. García-García, A. Redin, C. Muñoz-Almagro);; Institut de recerca Sant Joan de Déu, Esplugues de Llobregat (J.J. García-García, C. Muñoz-Almagro);; Hospital Vall d’ Hebron, Barcelona (S. González-Peris, B. Viñado);; Hospital HM Nens, Barcelona (A. Díaz-Conradi);; Universitat Internacional de Catalunya, Sant Cugat del Vallés, Spain (C. Muñoz-Almagro)

**Keywords:** invasive pneumococcal disease, serotype, 13-valent pneumococcal conjugate vaccine, nonpharmaceutical measures, COVID-19, SARS-CoV-2, coronavirus disease, severe acute respiratory syndrome coronavirus 2, bacteria, viruses, respiratory infections, zoonoses, Catalonia, Spain

## Abstract

We analyzed the effect of COVID-19 on healthcare demand and invasive pneumococcal disease in children in Catalonia, Spain. Compared with 2018–2019, we noted large reductions in healthcare activities and incidence of invasive pneumococcal disease in 2020. These changes likely resulted from nonpharmaceutical measures implemented during the COVID-19 pandemic.

SARS-CoV-2 was identified in 2019, and the World Health Organization declared COVID-19 a pandemic on March 11, 2020. As of July 11, 2021, >186 million cases and >4 million deaths had been recorded (*1*).

The first imported case of COVID-19 in Catalonia, Spain, was reported on February 26, 2020. Endemic transmission was declared on March 14, when the government of Spain introduced a strict lockdown until May 11. Other mandates followed, such as mask use, physical distancing, and reducing frequency of social contacts to reduce disease transmission (*2*). The peak number of cases was recorded in April 2020; cases subsequently declined and then occurred in epidemic waves. In 2020, a total of 356,724 cases and 8,723 deaths were reported in Catalonia (*3*).

Measures to reduce COVID-19 transmission have been associated with a reduction in diseases caused by respiratory pathogens, such as invasive pneumococcal disease (IPD) (*4*). IPD caused by *Streptococcus pneumoniae* has high rates of severe illness and death, especially in the very old and very young. In Catalonia, IPD incidence in children <5 years of age was 29.1/100,000 population in 2018, and 74.4% of cases were caused by serotypes not included in the 13-valent pneumococcal conjugate vaccine (PCV13) (*5*). Vaccine coverage in children was 92.9% in 2019. We assessed the effect of COVID-19 on the demand for care of IPD in children in 2020 compared with 2018–2019.

## The Study

We investigated IPD cases identified during 2018–2020 in 3 pediatric hospitals, Sant Joan de Déu, Vall d’Hebron, and HM Nens, which serve 521,463 children <18 years of age, 32% of pediatric patients in Catalonia. A confirmed case of IPD was defined as isolation or detection of *S. pneumoniae* DNA by PCR from a normally sterile site. Data collected were number of emergency department (ED) visits and admissions; requests for sterile cultures as blood, cerebrospinal fluid, and pleural fluid; requests for PCR for pneumococcus; confirmed cases of IPD; and serotype distribution.

We calculated mean incidence rates per 100,000 person-years by using population served by the 3 hospitals each year. We compared incidence rates in 2018–2019 with 2020 rates by calculating incidence rate ratio (IRR) and 95% CI annually, by quarters and age groups (0–4 and 5–17 years). We expressed percentage change in IRR according to the formula (1–IRR) × 100. We performed analysis by using R version 3.5.0 (The R Project for Statistical Computing, https://www.r-project.org).

Total numbers of visits to EDs were 225,031 in 2018, 229,256 in 2019, and 148,637 in 2020; total numbers of hospital admissions were 11,421 in 2018, 11,206 in 2019, and 8,423 in 2020. Compared with mean incidence in 2018–2019, ED visits declined by 35% in 2020, and hospital admissions declined by 26% (Table 1). The number of cultures was reduced in 2020 by 6%, and the number of requested PCR tests specific for *S. pneumoniae* declined by 23%, predominantly in children 0–4 years of age (23%).

**Table 1:** Healthcare activity and IPD incidence by age group, Catalonia, Spain, 2018–2019 and 2020*

Variable	No. cases (incidence, cases/100,000 population)		IRR (95% CI)	p value
	Mean 2018–19	2020		
All ages				
Emergency department visits	227,148 (43,661.3)	148,637 (28,437.6)	0.65 (0.64–0.66)	<0.0001
Hospital admissions	11,313 (2,174.5)	8,423 (1,611.5)	0.74 (0.72–0.76)	<0.0001
Samples for culture, HSJD	7,489 (1,439.5)	7,106 (1,359.5)	0.94 (0.91–0.98)	0.001
Samples for PCR, HSJD and HVH	641 (123.2)	497 (95.1)	0.77 (0.69–0.87)	<0.0001
IPD cases	57 (11.0)	20 (3.8)	0.35 (0.21–0.57)	<0.0001
PCV13 serotypes	25 (4.8)	10 (1.9)	0.40 (0.18–0.82)	0.01
Serotype 3	17 (3.3)	9 (1.7)	0.53 (0.22–1.17)	0.07
Non-PCV13 serotypes	29 (5.6)	10 (1.9)	0.34 (0.17–0.70)	0.003
0–4 y				
Emergency department visits	108,757 (93,016.7)	68,684 (60,617.9)	0.65 (0.64–0.66)	<0.0001
Hospital admissions	6,519 (5,575.5)	4,256 (3,756.2)	0.67 (0.65–0.70)	<0.0001
Samples for culture, HSJD	ND	ND	NA	NA
Samples for PCR, HSJD and HVH	459 (392.6)	342 (301.8)	0.77 (0.67–0.88)	0.0002
IPD cases	44 (37.6)	15 (13.2)	0.35 (0.19–0.62)	0.0001
PCV13 serotypes	18 (15.4)	8 (7.1)	0.46 (0.19–1.04)	0.06
Serotype 3	12 (10.3)	8 (7.1)	0.69 (0.27–1.69)	0.42
Non-PCV13 serotypes	25 (21.4)	7 (6.2)	0.29 (0.12–0.67)	0.002
5–17 y				
Emergency department visits	118,391 (29,353.5)	79,953 (19,530.7)	0.66 (0.65–0.67)	<0.0001
Hospital admissions	4,794 (1,188.6)	4,167 (1,017.9)	0.86 (0.82–0.89)	<0.0001
Samples for culture. (HSJD	ND	ND	NA	NA
Samples for PCR. HSJD and HVH	182 (45.1)	155 (37.9)	0.84 (0.68–1.04)	0.11
IPD cases	13 (3.2)	5 (1.2)	0.38 (0.13–1.06)	0.06
PCV13 serotypes	7 (1.7)	2 (0.5)	0.28 (0.06–1.36)	0.17
Serotype 3	5 (1.2)	1 (0.2)	0.20 (0.02–1.69)	0.21
Non-PCV13 serotypes	4 (1.0)	3 (0.7)	0.74 (0.16–3.30)	0.71

*HSJD, Hospital Sant Joan de Dèu; HVH, Hospital Vall Hebron; IPD, invasive pneumococcal disease; IRR, incidence rate ratio; NA, not applicable; ND, not done; PCV13, 13-valent pneumococcal conjugate vaccine.

**Figure 1:**
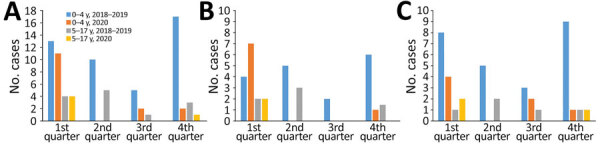
Invasive pneumococcal disease cases by quarter, age group, and year, Catalonia, Spain. A) Global cases; B) 13-valent pneumococcal conjugate vaccine serotypes; C) non–13-valent pneumococcal conjugate vaccine serotypes.

**Figure 2:**
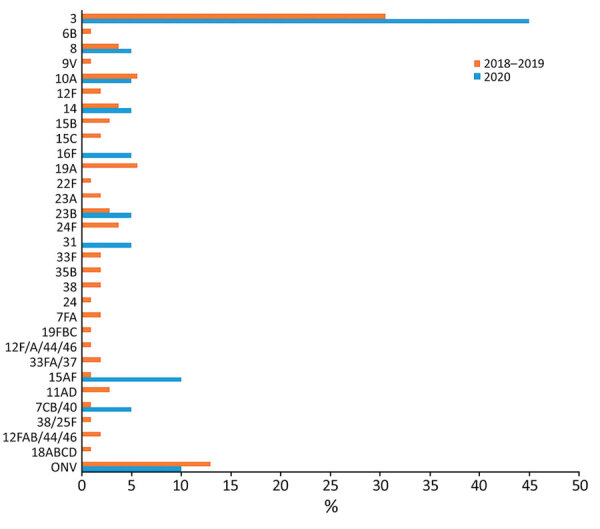
Distribution of invasive pneumococcal disease serotypes, Catalonia, Spain, 2018–2019 and 2020

IPD incidence per 100,000 person-years was 11 in 2018–2019 and 3.8 in 2020, a reduction of 65%; this same reduction was observed in the 0–4-year age group in 2020 (Table 2). Reduction of IPD incidence in 2020 was greater in the second and fourth quarter; no IPD cases were reported in the second quarter of 2020. Incidence per 100,000 person-years of IPD caused by PCV13 serotypes was 4.8 in 2018–2019 and 1.9 in 2020; IPD caused by non-PCV13 serotypes was 5.6 in 2018–2019 and 1.9 in 2020 (Table 1; Figure 1). Serotype 3 was the most frequent serotype in 2018–2019 (30.6%) and 2020 (45%) (Figure 2).

**Table 2:** Healthcare activity and IPD incidence by quarter, Catalonia, Spain, 2018–19 and 2020*

Variable	No. cases (incidence, cases/100,000 population)		IRR (95% CI)	p value
	Mean 2018–2019	2020		
1st quarter				
Emergency department visits	61,590 (11,838.5)	54,430 (10,413.7)	0.88 (0.87–0.89)	<0.0001
Hospital admissions	3,049 (586.1)	2,785 (532.8)	0.91 (0.86–0.96)	0.0003
Samples for culture, HSJD	1,968 (378.3)	2,192 (419.4)	1.11 (1.04–1.18)	0.0009
Samples for PCR, HSJD and VH	185 (35.6)	182 (34.8)	0.98 (0.80–1.20)	0.84
IPD cases	17 (3.3)	15 (2.9)	0.88 (0.43–1.77)	0.72
PCV13 serotypes	7 (1.3)	9 (1.7)	1.28 (0.47–3.62)	0.64
Serotype 3	5 (1.0)	8 (1.5)	1.59 (0.51–5.35)	0.43
Non-PCV13 serotypes	10 (1.9)	6 (1.1)	0.60 (0.20–1.65)	0.33
2nd quarter				
Emergency department visits	55,519 (10,671.6)	23,025 (4,405.2)	0.41 (0.40–0.42)	<0.0001
Hospital admissions	2,772 (532.8)	1,670 (319.5)	0.60 (0.56–0.64)	<0.0001
Samples for culture, HSJD	1,891 (363.5)	1,633 (312.4)	0.86 (0.80–0.92)	<0.0001
Samples for PCR, HSJD and VH	141 (27.1)	107 (20.5)	0.76 (0.59–0.97)	0.03
IPD cases	15 (2.9)	0	NA	<0.0001
PCV13 serotypes	8 (1.5)	0	NA	0.008
Serotype 3	6 (1.2)	0	NA	0.03
Non-PCV13 serotypes	6 (1.2)	0	NA	0.03
3rd quarter				
Emergency department visits	44,594 (8,571.6)	34,933 (6,683.5)	0.78 (0.77–0.79)	<0.0001
Hospital admissions	2,171 (417.3)	1,810 (346.3)	0.83 (0.78–0.88)	<0.0001
Samples for culture, HSJD	1,789 (343.9)	1,618 (309.6)	0.90 (0.84–0.96)	0.002
Samples for PCR, HSJD and VH	112 (21.5)	86 (16.5)	0.76 (0.58–1.01)	0.06
IPD cases	6 (1.2)	2 (0.4)	0.33 (0.05–1.57)	0.18
PCV13 serotypes	2 (0.4)	0	–	0.25
Serotype 3	1 (0.2)	0	–	0.50
Non-PCV13 serotypes	3 (0.6)	2 (0.4)	0.66 (0.11–3.97)	0.65
4th quarter				
Emergency department visits	65,445 (12,579.5)	36,249 (6,935.3)	0.55 (0.54–0.56)	<0.0001
Hospital admissions	3,321 (638.4)	2,158 (412.9)	0.65 (0.61–0.68)	<0.0001
Samples for culture, HSJD	1,841 (353.9)	1,663 (318.2)	0.90 (0.84–0.96)	0.002
Samples for PCR, HSJD and VH	203 (39.0)	122 (23.3)	0.60 (0.48–0.75)	<0.0001
IPD cases	19 (3.7)	3 (0.6)	0.16 (0.04–0.49)	0.001
PCV13 serotypes	8 (1.5)	1 (0.2)	0.12 (0.01–0.78)	0.02
Serotype 3	5 (1.0)	1 (0.2)	0.20 (0.01–1.44)	0.22
Non-PCV13 serotypes	10 (1.9)	2 (0.4)	0.20 (0.03–0.82)	0.02

*HSJD, Hospital Sant Joan de Dèu; HVH, Hospital Vall Hebron; IPD, invasive pneumococcal disease; IRR, incidence rate ratio; NA, not applicable; PCV13, 13-valent pneumococcal conjugate vaccine.

## Conclusions

The lockdown during the first months of the COVID-19 pandemic in 2020, together with social distancing measures, reduced mobility, and limits on the number of persons at social gatherings, had a positive effect on preventing IPD transmission in children and on indicators of healthcare activity. Overall reduction in IPD incidence was observed throughout 2020 compared with incidence for 2018–2019. No IPD cases were detected in the second quarter of 2020, coinciding with the lockdown, and a reduction of 84% was observed in the fourth quarter, coinciding with intensifying containment measures after the second wave of COVID-19 (*6*).

The percentage reduction in IPD cases in 2020 was similar in children <5 years of age (65%) and those 5–17 years of age (62%), although in the older group the reduction was not statistically significant because very few cases occurred in 2020. Other authors have described reductions in IPD during the COVID-19 pandemic. A prospective analysis from 26 countries found reductions of IPD of 68% at 4 weeks and of 82% at 8 weeks (*7*). In Hong Kong, observed IPD cases declined by 74.7% in 2020 compared with 2015–2019 (*8*). Some authors have stated that during 2020 no campaign occurred to increase pneumococcal vaccination and no other changes in practice affecting diagnosis or notification requirements for IPD were enacted that would explain reductions in incidence (*9*).

Serotype 3 was the most frequent serotype in the 2 periods (30.6% in 2018–2019 and 45% in 2020), and no significant reduction was detected. Similar results were observed by Teng et al. (*8*): 52.9% of cases in 2015–2019 and 45.7% in 2020 were serotype 3. We observed reduction in IPD incidence in 2020 compared with 2018–2019 in PCV13 (60%) and non-PCV13 (66%) serotypes.

A systematic review of 15 studies (*10*) concluded that nonpharmaceutical interventions could delay the introduction of influenza virus and are therefore effective in controlling influenza epidemics. In Catalonia, the active surveillance system for influenza and other acute respiratory infections found that influenza epidemic activity in the 2019–20 season had a short duration of 8 weeks (weeks 3–11) (*11*). Other authors recorded a similar situation; influenza and respiratory syncytial virus incidence declined sharply, and the season in 2020 was brief and ended rapidly compared with previous years (*12*). Viral infections might create favorable conditions in nasopharyngeal mucosa for invasive, colonizing pneumococcus causing IPD, so reduced influenza transmission during the pandemic might also have contributed to the reduction in IPD (*8*).

We found a reduction in ED visits (35%) and hospital admissions (26%) for IPD in 2020 compared with 2018–2019. Declines were greatest in the second quarter (59% for ED visits, 40% for hospital admissions), followed by the fourth quarter (45% for ED visits, 35% for hospital admissions), coinciding with the total lockdown and more stringent public health measures adopted because of the second epidemic wave in this setting (*6*). The number of cultures and specific requests for *S. pneumoniae* PCR declined less than the number of ED visits, hospital admissions, and IPD incidence in 2020. Increased public awareness of adequate individual use of nonpharmaceutical protective measures and social distancing measures had an effect on reducing incidence of IPD and other respiratory infections (*13*).

One limitation of our study is that the data analyzed came from just 32% of pediatric patients in Catalonia treated in 3 pediatric reference hospitals. However, the hospitals were reference hospitals; therefore, we believe these data are representative of the pediatric population in Catalonia. In addition, not all patients were tested during the first wave, so the exact incidence of SARS-CoV-2 infection in the first months of the pandemic is unknown. A strength of the study is that data were collected in a similar way throughout the study.

In summary, the reduction in ED visits and hospital admissions in 2020 compared to 2018–2019 in Catalonia was greater than the reduction in requests for culture and PCR specific for *S. pneumoniae.* The reduction in IPD incidence was more marked during the second quarter of 2020, coinciding with COVID-19 lockdowns.
